# Diagnostische und prädiktive Marker in der Harntraktzytologie

**DOI:** 10.1007/s00292-022-01053-9

**Published:** 2022-02-08

**Authors:** Tatjana Vlajnic, Lukas Bubendorf

**Affiliations:** Pathologie, Institut für medizinische Genetik und Pathologie, Universitätsspital Basel, Schönbeinstr. 40, 4031 Basel, Schweiz

**Keywords:** Differenzialdiagnose, Hochdurchsatz-Nukleotidsequenzierung, Immunhistochemie, Immuncheckpoint-Inhibitoren, Urologische Tumoren, Differential diagnosis, High-throughput nucleotide sequencing, Immunohistochemistry, Immune checkpoint inhibitors, Urologic neoplasms

## Abstract

In der Routinediagnostik spielt die Mehrfach-Fluoreszenz-in-situ-Hybridisierung (FISH) nach wie vor die führende Rolle in der Abklärung unklarer Atypien in der Harntraktzytologie. Die Paris-Klassifikation (The Paris System, TPS) bildet eine wichtige Grundlage zur gezielten Indikationsstellung der FISH und untermauert die Bedeutung der morphologischen Korrelation für eine integrative Diagnosestellung. Die Next-Generation-Sequencing-Technologie, welche durch gleichzeitigen Nachweis multipler genetischer Alterationen eine hohe Sensitivität erzielt, wird in naher Zukunft auch in der Harntraktzytologie Anwendung finden.

## Abstract (en)

Multiprobe fluorescence in situ hybridization (FISH) still remains the gold standard for clarifying inconclusive atypia in urinary cytology in daily routine practice. The Paris Classification System (The Paris System, TPS) provides an important basis for the specific indication of FISH and emphasizes the importance of morphological correlation for an integrative approach to diagnosis. Next-generation sequencing technology in urinary specimens, which is highly sensitive for simultaneous detection of multiple genetic alterations, is also likely to play a diagnostic role in the near future.

Die Zytologie besitzt einen zentralen Stellenwert bei der Abklärung von Erkrankungen des Harntraktes und in der Nachsorge von Patienten mit bekannten urothelialen Neoplasien. Ihre Stärke liegt zum einen in der hohen Zuverlässigkeit für die Diagnose eines potenziell lebensbedrohlichen high-grade Urothelkarzinoms (HGUC), zum anderen in der relativ einfachen und wenig invasiven Methodik zur Gewinnung von Untersuchungsmaterial, entweder aus der Spülflüssigkeit im Rahmen einer Endoskopie oder auch aus Spontanurin.

Vor einigen Jahren wurde durch die Einführung der Paris-Klassifikation (The Paris System, TPS) eine internationale Standardisierung in der Befundung von Urinzytologien ermöglicht [1, 2]. Neben der zuverlässigen Diagnose bzw. dem Ausschluss eines HGUC liegt ein weiterer Schwerpunkt dieser Klassifikation darin, die Häufigkeit der bislang uneinheitlich genutzten Diagnose von „Atypien“ zu verringern und so die Aussagekraft der Urinzytologie zu verbessern. Basierend auf den strikt definierten morphologischen Kriterien der Paris-Klassifikation lassen sich eindeutig negative oder eindeutig maligne Befunde in der Mehrzahl der Fälle als solche erkennen. Ein Teil bleibt dennoch morphologisch unklar und erfordert Zusatzmethoden zur definitiven Klärung (Tab. 1). Trotz erheblicher und kontinuierlicher Anstrengungen über die letzten beiden Jahrzehnte haben sich bisher nur wenige Biomarker und Methoden der Urinzytologie in der klinische Routine zur verbesserten Diagnostik und/oder Verlaufskontrolle als nützlich erwiesen [3–5]. Zunehmend wächst auch die Nachfrage nach prädiktiven Markern beim Urothelkarzinom in Zusammenhang mit personalisierter Medizin.

**Table 1:** 

*Hauptkriterium* (erforderlich):
Nichtsuperfizielle und nichtdegenerativ veränderte Urothelien mit Kern-Plasma-Relation > 0,5
*Nebenkriterien* (mindestens eines):
Geringe Hyperchromasie
Kernmembran: Unregelmäßigkeiten (Kontur und Dicke)
Chromatin: irregulär, vergröbert, verklumpt

## Immunzytochemie

**Figure 1:**
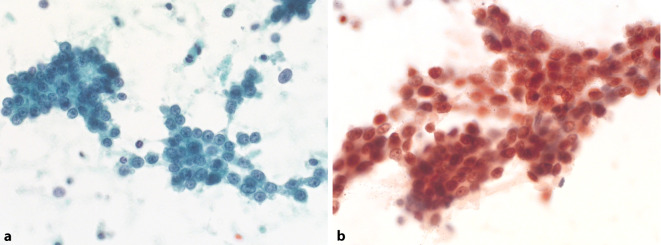

Immunzytochemische Untersuchungen in der Urinzytologie wurden im diagnostischen Alltag bislang vor allem für differenzialdiagnostische Fragestellungen bzw. für die Abgrenzung des Urothelkarzinoms von Manifestationen anderer Tumoren eingesetzt. Grundsätzlich sollte die Immunzytochemie direkt an Ausstrichpräparaten/Zytospins etabliert werden, da eine Anfertigung von Zellblöcken in der Urinzytologie unüblich ist. Zu den gebräuchlichsten Markern zum Nachweis urothelialen Ursprungs von malignen Epithelzellen in der Harntraktzytologie zählen GATA3, CK7, CK20 und p40 oder p63 [6]. Die weitaus häufigste Differenzialdiagnose ist das Adenokarzinom der Prostata. Falls die typischen morphologischen Charakteristika wie azinäre Strukturen und prominente Nukleolen fehlen, kann NKX3.1 als relativ spezifischer und sensitiver Marker für Karzinome prostatischen Ursprungs eingesetzt werden ([7]; Abb. 1). Im Gegensatz zum Urothelkarzinom sind Adenokarzinome der Prostata zudem praktisch immer negativ für die urothelialen Marker CK7, GATA3 und p40/p63 [8]. Basalzellkarzinome des Prostata mit aberranter p63-Expression sind eine Rarität [9]. Als weiterer spezifischer Marker für Prostatakarzinome gilt PSA, welches jedoch bei wenig differenzierten Prostatakarzinomen lediglich schwach oder gar nicht exprimiert wird. Falls nur wenige Präparate für eine immunzytochemische Untersuchung zur Verfügung stehen, ist eine Priorisierung der beiden Marker NKX3.1 und CK7 sinnvoll. CK7 wird in Urothelkarzinomen praktisch immer (87–100 %) exprimiert [10]. Eine fokale CK7-Positivität wurde in bis zu 10 % der Adenokarzinome der Prostata beschrieben [11]. Nach unserer Erfahrung wird dieser Marker bei Prostatakarzinomen jedoch nie diffus exprimiert. Eine einfache immunhistochemische Bestimmung des molekularen Subtyps (v. a. basal und luminal) mittels geeigneter Marker (z. B. CK5/6 oder p63 und CK20 oder GATA3) wäre zwar grundsätzlich interessant und potenziell relevant, gilt aber derzeit noch nicht als Standard [12–14] und hätte in der Harntraktzytologie nur eine untergeordnete oder keine praktische Bedeutung.

**Figure 2:**
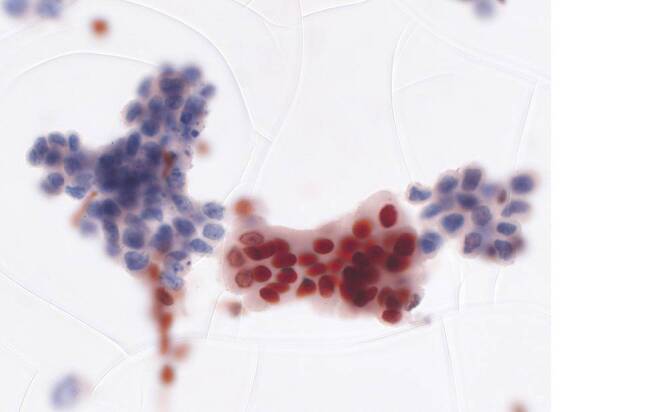

**Figure 3:**
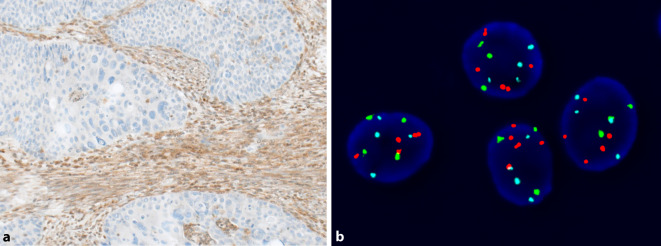

Eine weitere Anwendung der Immunzytochemie wurde erst kürzlich auf potenzielle prädiktive Marker erweitert. Inaktivierende Mutationen im *ARID1A*-Gen wurden mit reduziertem Ansprechen auf Bacillus-Calmette-Guérin(BCG)-Therapie in Verbindung gebracht [15]. Eine Studie zeigte, dass ein Expressionsverlust von ARID1A in der Immunzytochemie als verlässlicher Surrogatmarker für *ARID1A*-Mutationen dient ([16]; Abb. 2). Die immunzytochemische Untersuchung der PD-L1-Expression zur Selektion von Patienten für eine Behandlung mit Immuncheckpoint-Inhibitoren (ICI) ist an Papanicolaou-gefärbten zytologischen Präparaten oder Zellblockpräparaten grundsätzlich möglich [17]. Allerdings können an zytologischen Präparaten nur Tumorzellen gewertet werden, sodass nur eine Bestimmung des PD-L1-Tumor-Proportion-Score (TPS) möglich ist. Eine Aussage über die Expression an Immunzellen und den PD-L1-Combined-Positive-Score (CPS) ist nur eingeschränkt möglich, da sich die tumorassoziierten Immunzellen ohne Gewebekontext in der Zytologie nicht auswerten lassen. Bei einem Anteil PD-L1-positiver Tumorzellen (TPS) von > 10 %, liegt der CPS jedoch ungeachtet der tumorassoziierten Immunzellen definitionsgemäß ebenfalls bei über 10, was mit einem Ansprechen auf den Immuncheckpoint-Inhibitor (ICI) Pembrolizumab assoziiert ist. Die PD-L1-Testung in der Harntraktzytologie stellt im Alltag aber keine Notwendigkeit dar, da bei fortgeschrittenen Urothelkarzinomen praktisch immer ausreichend histologisches Material für eine PD-L1-Testung mit Bestimmung des CPS zur Verfügung steht [18]. Ein Verlust der MTAP-Expression als Surrogatmarker für eine homozygote 9p21-Deletion wurde kürzlich als potenziell interessanter negativer prädiktiver Marker für das Ansprechen auf ICI bei verschiedenen malignen Tumoren inkl. dem Urothelkarzinom vorgeschlagen [19]. Auch hier dürfte in der Zukunft die Untersuchung an Gewebeproben im Vordergrund stehen, zumal ethanolfixierte zytologische Präparate für die MTAP-Immunzytochemie nach unserer Erfahrung ungeeignet sind (Abb. 3).

## Molekulare Diagnostik mittels Mehrfach-Fluoreszenz-in-situ-Hybridisierung

**Figure 4:**
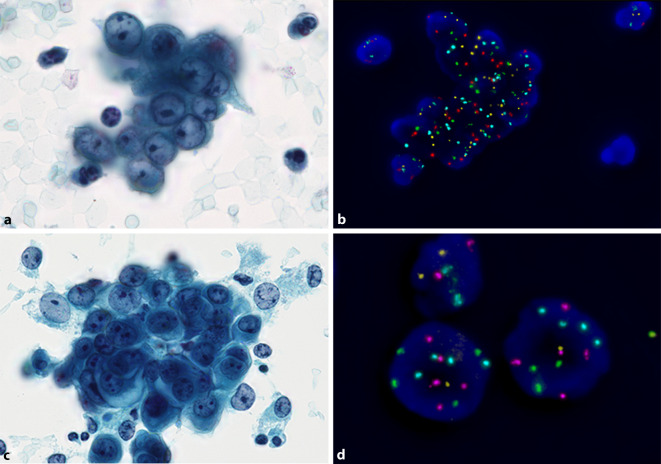

Für den Nachweis chromosomaler Aberration an zytologischen Präparaten eignet sich am besten eine Fluoreszenz-in-situ-Hybridisierung (FISH). Der kommerziell erhältliche und von der U.S: Food and Drug Administration (FDA) zugelassene Mehrfach-FISH-Test UroVysion™ (Abbott Laboratories, Abbott Park, IL, USA) bleibt nach wie vor eine der am besten etablierten Methoden zur Abklärung von Atypien in der Urinzytologie [20–23]. Dieser FISH-Test besteht aus 4 Sonden für die Chromosomen 3, 7, 17 und den 9p21-Lokus. Ein ähnlicher Quadrupel-FISH-Test eines anderen Herstellers ist verfügbar, wurde bisher aber nicht in größeren publizierten Studien verwendet (ZytoLight® Bladder Cancer Quadruple Color Probe, ZytoVision GmbH, Bremerhaven, Deutschland). Der Nachweis von unbalancierten numerischen chromosomalen Aberrationen in mindestens 2 Chromosomen und/oder ein kompletter oder relativer Verlust von 9p21 (entsprechend einer homozygoten oder heterozygoten 9p21-Deletion) sind diagnostisch für eine urotheliale Neoplasie und schließen reaktive Veränderungen mit praktischer Sicherheit aus [24]. Eine Ausnahme stellt das tetraploide oder oktaploide Muster dar (jeweils 4 bzw. 8 Signale von jeder Sonde), das auch bei ausgeprägt reaktiv veränderten Urothelien vorkommen kann. Dessen Nachweis sollte deshalb, insbesondere wenn nur in wenigen Zellen, nicht als eindeutig positives FISH-Resultat interpretiert werden [24]. Sensitivität und Spezifität dieser Untersuchung hängen stark von Erfahrung und Expertise des Untersuchers und von der zytologischen Diagnose ab. Dabei bieten die Paris-Kategorien eine wichtige Grundlage zur gezielten Indikationsstellung. Vor deren Hintergrund lässt sich die FISH-Untersuchung vor allem bei den Kategorien AUC (atypische urotheliale Zellen) und SHGUC (Verdacht auf high-grade Urothelkarzinom) sinnvoll in den diagnostischen Algorithmus implementieren ([24, 25]; Abb. 4). Wir haben kürzlich gezeigt, dass unsere Zytologien mit AUC oder SHGUC gemäß TPS in jeweils 46 % bzw. 84 % FISH-positiv waren, was in diesen Fällen für eine urotheliale Neoplasie und gegen reaktive Veränderungen spricht [25]. Somit ist unter Berücksichtigung der Morphologie und des FISH-Resultates oft eine zuverlässige Unterscheidung zwischen reaktiven Urothelveränderungen und einer urothelialen Neoplasie möglich. Entscheidend für die hohe Aussagekraft der FISH ist dabei eine gezielte Untersuchung atypischer Zellen. Insbesondere wenn wenige atypische Zellen zwischen reichlich normalen Urothelien untermischt sind, ist eine Untersuchung mittels automatisierter Relokalisationssoftware hilfreich, wobei die Koordinaten der Zielzellen vor der Hybridisierung und Interpretation durch Personen mit ausreichender Zytologieerfahrung markiert werden [26].

Tumoren mit einer homozygoten 9p21-Deletion scheinen gemäß einer kürzlich publizierten Arbeit häufig primär resistent auf eine Behandlung mit ICI zu sein [19]. Somit könnte die UroVysion™-FISH-Untersuchung an ethanolfixierten zytologischen Präparaten in Zukunft auch eine prädiktive Bedeutung erlangen. Wie bereits erläutert, kann an histologischen Proben der immunhistochemische Verlust der MTAP-Expression als Surrogatmarker für eine homozygote 9p21-Deletion verwendet werden (Abb. 3).

## Zukunftsausblick – Molekulare Diagnostik mittels Next Generation Sequencing

Grundsätzlich lassen sich molekulare Analysen unterteilen in solche, die an zytologischen Präparaten bzw. zytologiebasiert erfolgen sowie in flüssigkeitsbasierte Methoden zur Detektion von Proteinen oder molekularen Veränderung im Urin oder Blut ohne morphologische Korrelation [24]. Letztere werden in der Regel außerhalb eines zytologischen Labors durchgeführt und sind deshalb nicht Thema dieses Beitrags.

Verschiedene genetische Veränderungen wie wiederkehrende Mutationen und Kopienzahlveränderungen lassen sich in > 95 % der low-grade und high-grade Urothelkarzinome nachweisen und stellen somit das Rationale zur ergänzenden diagnostischen Testung dar [27–29]. Die Gene *TERT* (21–73 %), *FGFR3* (13–45 %), *PIK3CA* (20–23 %), *KDM6A* (26–48 %) und *ARID1A* (10–20 %) sind beim Urothelkarzinom je nach Stadium und Lokalisation besonders häufig mutiert und daher in molekularen Assays oft vertreten [30, 31]. Einige der häufigen Genmutationen haben prognostische Implikationen und wurden z. B. mit Tumorgrad und Stadium bei Präsentation assoziiert, andere korrelieren mit Therapieansprechen [5]. Insbesondere *FGFR*-Alterationen, die in ca. 15 % der fortgeschrittenen Urothelkarzinome vorkommen, gelten als prädiktiv für das Ansprechen auf FGFR-Inhibitoren [14]. Dank des technischen Fortschritts in den letzten Jahren ist die klassische Sanger-Sequenzierung zunehmend durch neue Sequenziertechniken (Next Generation Sequencing, NGS) ersetzt worden. NGS erlaubt nicht nur den Nachweis von Mutationen und Fusionen, sondern auch von epigenetischen Veränderungen (DNA-Methylierung) und Kopienzahlveränderungen und bietet den Vorteil, dass diese gleichzeitig nachgewiesen werden können. Allerdings ist anzumerken, dass RNA-basierte Untersuchungen zum Nachweis von Genfusionen aus zytologischen Präparaten je nach Fixierungsmethode und Menge an Tumorzellen schwieriger sein können als aus Paraffinblöcken. NGS-basierte Analysen von zytologischen Präparaten des Harntrakts versprechen somit eine hohe Sensitivität und werden zukünftig wahrscheinlich eine Anwendung im diagnostischen Alltag finden. Als gutes Beispiel dient der UroSEEK-Assay am Urin, bei dem 11 ausgewählte Gene auf Mutationen oder Veränderungen der Kopienzahl untersucht werden. Dieser Test wurde für die Nachkontrolle von Patienten mit resezierten Urothelkarzinomen und für die Abklärung von zytologischen Atypien entwickelt [28, 32, 33]. In einer retrospektiven Studie zeigte er vor allem bei low-grade nichtinvasiven papillären Urothelkarzinomen eine hohe Sensitivität im Vergleich zur Zytologie [32]. Allerdings ist für die Diagnose von low-grade nichtinvasiven papillären Urothelkarzinomen, die zystoskopisch gut sichtbar sind, die Indikation fragwürdig, da der Test in Relation zu den hohen Kosten wenig klinisch relevante Zusatzinformationen liefert. In einer Follow-up-Kohorte von Patienten mit „Atypien“ in der Harntraktzytologie (AUC- und SHGUC-Kategorien zusammengenommen) erwies sich der UroSEEK-Assay mit einer Sensitivität von 74 % und einem negativen prädiktiven Wert von 53 % als weniger robust [33]. Die wichtigste Voraussetzung für die NGS-Analyse ist eine ausreichende Anzahl bzw. ein relativer Anteil an Tumorzellen von mind. 2 % in der zytologischen Probe. Daher bieten sich insbesondere zellreiche Urinzytologieproben für eine NGS-Testung an. In einer kürzlich veröffentlichten Übersichtsarbeit zur Rolle des NGS in der Urinzytologie wurde vorgeschlagen, die Urinzytologie als Triage zu benutzen. Somit ließe sich beurteilen, welche Präparate für eine direkte NGS-Testung geeignet sind und bei welchen die Tumorzellen, z. B mittels Laser-Mikrodissektion, angereichert werden müssten [5]. Obwohl NGS zweifellos eine vielversprechende Methode in der Harntraktzytologie darstellt und mit weiteren technischen Fortschritten auf diesem Gebiet zu rechnen ist, sind umfassende Studien zur Festlegung der Wertigkeit und der optimalen Indikationen notwendig. Neben technischen Faktoren muss vor allem auch das Kosten-Nutzen-Verhältnis kritisch untersucht werden. Ökonomische Interessen an einer breiten Anwendung sollten dem medizinischen Zusatzgewinn im Vergleich zu einer standardisierten zytologischen Diagnose gegenübergestellt werden.

## Fazit für die Praxis

- Die Morphologie besitzt nach wie vor eine zentrale Bedeutung in der Harntraktzytologie und hat durch die standardisierte Befundung im Rahmen des Paris-Systems (TPS) an Bedeutung gewonnen.
- Die Immunzytochemie ist in ausgewählten Fällen nützlich für eine diagnostische Zuordnung von Tumorzellen und kann auch einen potenziell prädiktiven Wert besitzen.
- Die Fluoreszenz-in-situ-Hybridisierung ist bei unklaren Befunden der TPS-Kategorien AUC (atypische urotheliale Zellen) oder SHGUC (Verdacht auf high-grade Urothelkarzinom) diagnostisch hilfreich.
- Next Generation Sequencing ist eine erfolgversprechende Zusatzmethode für die Harntraktzytologie, deren praktischer Wert für die verschiedenen zytologischen Kategorien und klinischen Situationen aber kritisch hinterfragt und noch weiter untersucht werden muss.
